# Low-loss and dual-band filter inspired by glide symmetry principle over millimeter-wave spectrum for 5G cellular networks

**DOI:** 10.1016/j.isci.2022.105899

**Published:** 2022-12-27

**Authors:** Mohsen Karamirad, Negin Pouyanfar, Mohammad Alibakhshikenari, Changiz Ghobadi, Javad Nourinia, Chan Hwang See, Francisco Falcone

**Affiliations:** 1 Department of Electrical Engineering, Urmia University, Urmia, Iran; 2 Department of Signal Theory and Communications, Universidad Carlos III de Madrid, Leganés, 28911 Madrid, Spain; 3 School of Engineering and the Built Environment, Edinburgh Napier University, 10 Colinton Road, Edinburgh EH10 5DT, UK; 4 Department of Electric, Electronic and Communication Engineering and the Institute of Smart Cities, Public University of Navarre, and the Tecnologico de Monterrey, School of Engineering and Sciences, 31006 Pamplona, Spain

**Keywords:** Applied sciences, Broadband communication, Network

## Abstract

This paper focuses on designing a dual-band, bandpass filter configuration inspired by glide-symmetric structures in a single plane. Geometry configuration of elliptical slots on both sides of single substrate generally affects electromagnetic fields as well as rejection bands. Easy fabrication with misalignment avoidance during assembly procedure unlike conventional structures based on gap waveguide technology, make them appropriate to use in electromagnetic devices. Parametric study on dispersion characteristics is carried out in this article to find out how rejection-bands are offered through breaking the symmetry. A method for producing symmetry is also suggested, which may be helpful for reconfigurable devices. Moreover, equivalent circuit model is demonstrated to get insight of the mechanism of the presented glide symmetry scheme. The transmission frequency ranges of two passbands with center frequencies of 19.74 GHz and 28.233 GHz are shown by the measured and calculated S- parameters of five unit-cell structures.

## Highlights

• Dual-band filter is demonstrated over mm-wave spectrum for 5G cellular networks
• Low loss filter based on glide symmetry principle is realized
• The structure is parametrically analyzed for dual band feature of the filter

## Graphical abstract

**Figure:**
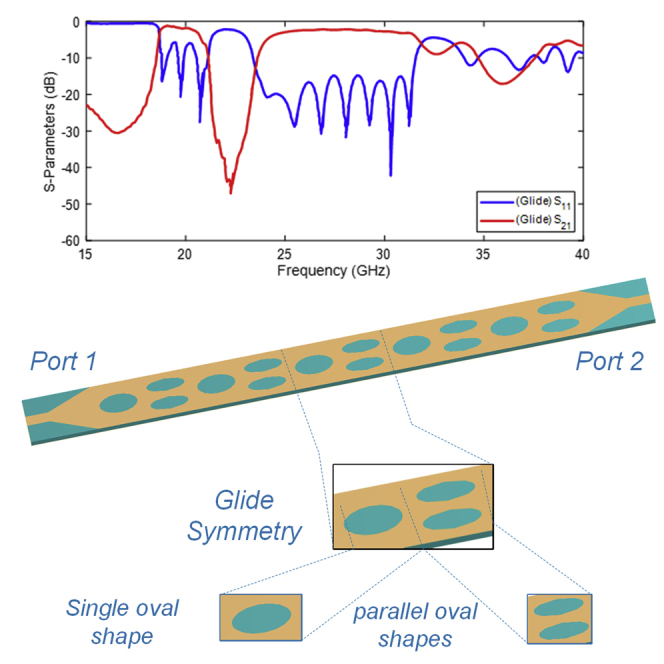

## Introduction

Fifth generation mobile networks have been increasingly demanded to overcome the previous mobile generations shortcomings such as bandwidth deficiencies because of increased number of users. Two practical frequency ranges have been allocated to the 5G cellular network in the Europe and United States. These frequency bands extend from 24.5 to 29.5 GHz and 37–43.5 GHz. Moreover, 28 GHz and 37–39 GHz frequency ranges are mainly allocated for the upcoming 5G wireless networks whereas satellite communication systems and navigation applications use 33 GHz as well.^1^^,^^2^^,^^3^ In addition, the progressive advances in higher frequencies have been led to infrastructures extensions for 60 GHz technology. Because most of the satellite communications operate in these frequency bands, the demand for designing sharp roll-off filters has been increased more than ever. As stated, because of the electronic devices and also deployed base station increment, design and manufacturing process is a serious challenge beside cost reduction.^4^ Furthermore, it should be considered that in higher frequencies, because of the small size of the devices their fragility increases as well as losses. A considerable attraction in using periodic structures to enhance the electromagnetic characteristics of practical microwave applications led to the recent exploration of greater symmetries.^5^^,^^6^^,^^7^^,^^8^^,^^9^ Recent investigations on higher symmetry structures (two-dimensional glide symmetries) have been reported, indicating remarkable capability for modifying the dispersion features of periodic structures.^10^^,^^11^ Transforming and mirroring a unit cell with respect to the glide plane yields periodic glide-symmetric structures.^12^ The dispersion of conventional structures can be reduced employing glide symmetry unit cell. However, to mitigate the increased losses at higher frequencies, it is useful to employ waveguide technology. For this purpose, a suitable alternative would be Gap-waveguides.^13^^,^^14^ In this regard, a structure based on glide-symmetry and gap-waveguide technologies is investigated in.^15^^,^^16^ Reduced periodic structure dispersion,^17^^,^^18^^,^^19^^,^^20^ increased equivalent refractive index,^21^^,^^22^^,^^23^ and increased band and attenuation of electromagnetic bandgaps^24^^,^^25^ have all been accomplished with the aid of glide symmetries. For fifth-generation (5G) communications, glide symmetry, for instance, has been suggested to create lens antennas,^26^^,^^27^ exploiting advantage of their capacity to provide an appropriate refractive index and low dispersion at desired frequency ranges.^10^ However, a major drawback of gap-waveguide technologies with respect to their design is complexity and high cost of manufacturing. Moreover, the integration of this technology to planar circuits is almost difficult. To prevail to limitation of 3D structures, microstrip lines can be an appropriate solution whose performance is similar to metallic waveguide.^28^ The glide symmetry topology has promising applications in various fields of flanges,^24^ leaky wave antennas,^29^ electromagnetic bandgap structures,^30^ and periodic surfaces.^31^^,^^32^ To better understanding the mechanism of the glide symmetry topology some approaches such as mode matching^33^ and equivalent circuit model^12^^,^^30^^,^^34^ have been applied.

In this paper, we propose a glide-symmetric structure in a single plane with ellipse slots as the main element on a dielectric substrate that serves as a support. Glide symmetry feature is achieved by placing the symmetry plane vertically while maintaining orthogonality with respect to the propagation direction. Further degrees of freedom can be introduced, when employing a dielectric substrate with varying dielectric constant values rather than an all-metal structure, such as the capability to alter or break the glide symmetry. The orientation, dimensions, and location of these ellipses indicate the electromagnetic features of the structure regarding dispersion and rejection-bands. The filter has a passband of 18.71 GHz–21.196 GHz and 23.54 GHz–31.86 GHz, with an insertion loss of 0.83 dB at 19.74 GHz and 1.3 dB at 28.233 GHz.

## Results

### Theory and design

#### Glide symmetry

**Figure 1:**
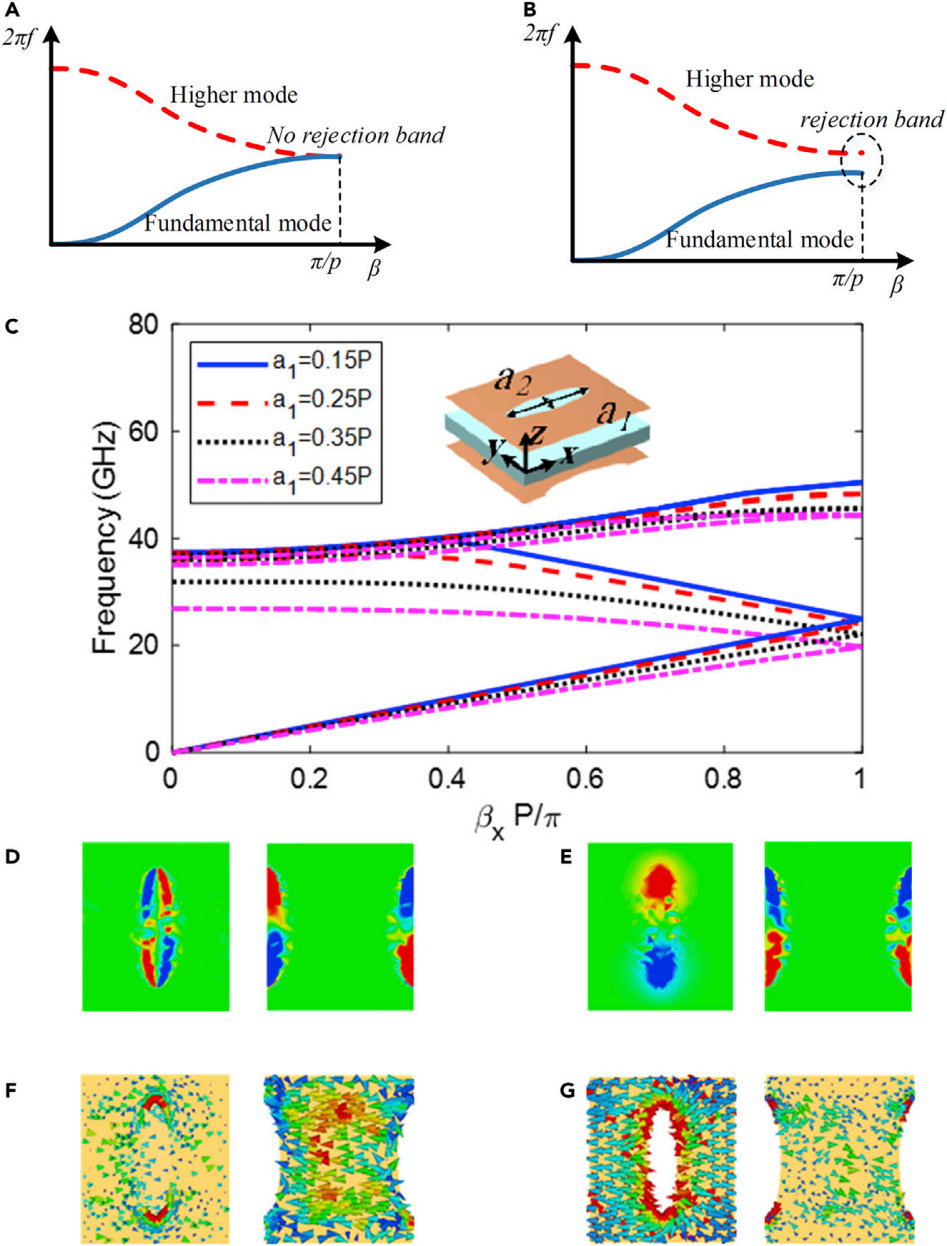
Dispersion analysis of the unit cell (A) No rejection-band configuration. (B) Rejection-band configuration. Dependence of radius of elliptical slot (a_1_) (inset picture) with $p_{x}=4\;mm$, $p_{y}=4.6\;mm$, $a_{2}/a_{1}=0.4$. Electric field ($\left|E_{y}\right|$) (D, E for mode 1) and surface current (F, G for mode 2) distribution at β=π/P . Left and right sides of each portion are top and bottom layers, respectively.

Recently, glide symmetry as a special issue in higher symmetries have been extensively attracted attention.^35^^,^^36^^,^^37^ A planar holey structure based on glide-symmetry is offered in^38^ to investigate electromagnetic properties. It is investigated in^19^^,^^39^ that stop/pass bands can be controlled by breaking the structure symmetries. Considering the advantages of breaking structure symmetry, we aim to achieve a rejection-band near the harmonic. In a periodic structure with periodicity of *p*, it can be defined as:

(Equation 1) $$BG_{1D}=\left\{ \begin{array}{c} x\to x+S.\frac{p}{2}\;0<S<1\\ y\to y\\ z\to -z \end{array}\right.$$

where *S* is defined as the glide factor and can get two values of S = 1 or S = 0 where the former indicates glide symmetry and the latter shows mirroring symmetry properties as well.^40^ Figure 1 shows the configuration of two examples. It is worth noting that suppression of rejection-band of glide symmetry structures can be explained using mode coupling mechanism. Accordingly, at β=π/p, the fundamental mode of forward wave and space harmonic of backward wave are coupled with each other along periodic cells. Accordingly, if coupling between two modes do not happen, a perfect cross is revealed. Otherwise, the dispersion diagram exhibits two new curves leading to a rejection-band at β=π/p. Therefore, it is necessary to make coupling be zero which is mathematically offered by expression (2).

(Equation 2) $$\int_{0}^{p}\int_{-\infty }^{\infty }\int_{-\infty }^{\infty }\left(E_{0}.E_{1}^{*}+E_{1}.E_{0}^{*}\right)dydzdx=0$$

where $E_{0}$ and $E_{1}$ are electric field of fundamental and space harmonic, respectively. For better express the electric field of glide symmetry, cylindrical coordinates are used. Thus, Equation 1, a condition for glide symmetry, is regenerated as E(r.θ.x)=E(r.−θ.x+p/2). In the following, Fourier series expansion of E(r.θ.x) is expressed in Equation 3.

(Equation 3) $$E\left(r.\theta .x\right)=\sum_{n=-\infty }^{\infty }\sum_{m=-\infty }^{\infty }E_{n.m}e^{-jm\theta }e^{-j2\pi nx/p}e^{-j\beta x}\;$$

According to,^41^ the Fourier coefficient $E_{n.m}\left(r\right)$ complies $E_{n.-m}\left(r\right)e^{-jn\pi }=E_{n.m}\left(r\right)$. Consequently, the fundamental and space harmonic satisfy $E_{0.-m}\left(r\right)=E_{0.m}\left(r\right)$ and $E_{1.-m}\left(r\right)={-E}_{1.m}\left(r\right)$, respectively. The indices m and n indicate the number of angular and axial space harmonic, respectively. Following this, the expression of total field is

(Equation 4) $$E_{0}=E_{0\cdot 0}e^{-j\beta x}+2\sum_{m=1}^{\infty }E_{0\cdot m}\cos\left(m\theta \right)e^{-j\beta x}$$

(Equation 5) $$E_{1}=-2j\sum_{m=1}^{\infty }E_{1.m}\;\sin\left(m\theta \right)e^{-j\beta x}e^{-\frac{j2\pi }{p}x}\text{.}$$

It is noted that the satisfaction of Equation 2 is achieved by Equations 4 and 5. The first harmonic of the forward wave is expressed by Equation 5. One should note that by substituting $e^{-j\beta x}$ with $e^{j\beta x}$, the first harmonic is obtained for backward wave. To sum up, the coupling between fundamental and space harmonic mode of forward and backward waves do not achieve for glide symmetry patterns. Therefore, a perfect cross is exhibited at β=π/p and the suppression of rejection-band is achieved. As stated above, the rejection-band can be controlled by breaking the glide symmetry.

### Glide-symmetric unit cell design

The investigated structures are made up of metallic patterned layers on top and bottom of a substrate layer. An elliptical slot and its glide symmetrical counterpart pattern are etched on top and bottom layers of the substrate, respectively. Propagation and the existence of rejection-bands vary as a result of changes in the shape and orientation of the ellipses as well as electromagnetic fields status within the dielectric substrate. The basic unit cell is depicted in the inset of Figure 1C with all of the parameters that characterize its electromagnetic behavior. The dispersion analysis was carried out using periodic conditions in the x- and y-directions of the unit cell. The propagation direction chosen in the analysis extends along the y-axis, where two periodicities generate glide symmetry. There is only one periodicity in the perpendicular direction (x-axis). CST microwave studio examined the proposed glide symmetric structure. Figure 1C depicts the dispersion relation for various elliptical pattern radii ($a_{1}$ and $a_{2}$) in the system with glide symmetry. As illustrated in Figure 1C, at the Brillouin zone boundary, the rejection-band between the fundamental mode (first propagation mode) and the second lowest-order mode (second mode) exhibits non-zero group velocity degeneracy. Furthermore, Figure 1C demonstrates that for a given value of the propagation constant in fundamental mode, the frequency decreases as the major radius of the elliptical slot increases. Moreover, Figure 1C shows that the equivalent refractive index of the proposed pattern increases as the radius of the elliptical pattern increases, indicating that the frequency at which the modes join tends to decreases at the Brillouin zone boundary.

To better understand the mechanism, the electric fields of modes 1 and 2 are shown in Figures 1D–1G. As illustrated in the figure, there is no overlap (zero coupling) and the rejection-band is suppressed at β=π/p. Figures 1D–1G also demonstrate that the E-field is oppositely presented on top and bottom layers and also, the symmetric (for first mode) and asymmetric (for second mode) directions of the resultant current vector confirm the zero coupling at β=π/p.

**Figure 2:**
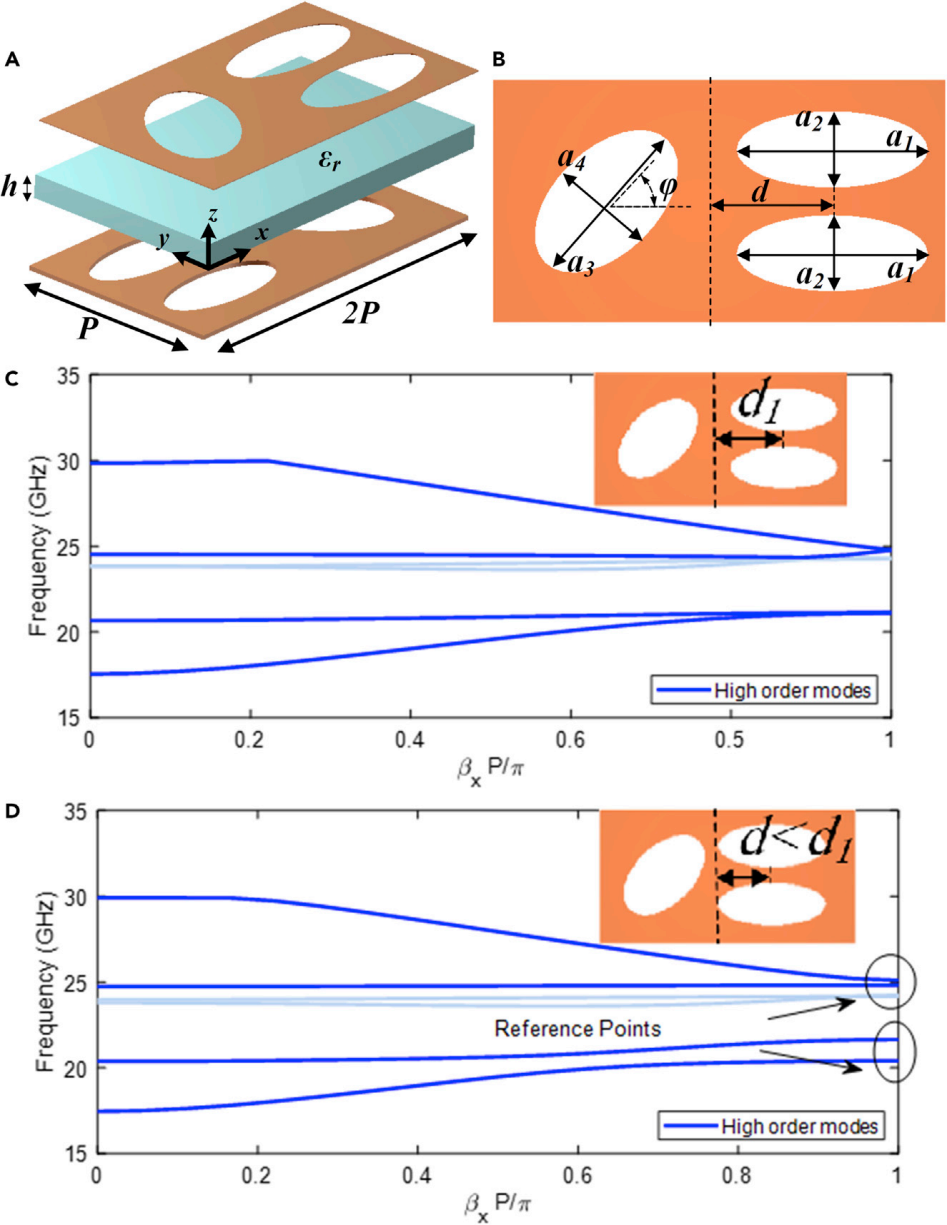
Dispersion analysis of the braided unit cell (A) Perspective view of the unit cell. (B) Top view of the unit cell. The parameters are detailed in the picture. (C) Glide and (D) none-glide symmetry configurations. Higher order modes are shaded.

The parameters affecting the dimension, position, orientation, and shape of the slots which are responsible for the overall characteristics of the proposed structure are depicted in Figure 2. The values of $a_{i}$ and $b_{i}$ (i = 1, 2, 3, 4) define the dimensions of the major and minor axes of the slot on the top and bottom layers, respectively. The parameters θ and ϕ=−θ define the inclination angle of the single ellipse on the top and bottom layers, respectively. A zero value indicates that the ellipses are horizontal (y-directed minor axis). Positive values of θ cause the single ellipse on the top layer to rotate counter-clockwise, where positive values of ϕcause the single ellipse on the bottom layer to rotate clockwise. Finally, the displacement parameter d can be used to change the distance between two parallel ellipses from the symmetrical plane. The ellipses are kept at a distance of $d=d_{1}$, whereas values less than $d_{1}$ indicate that the ellipses have moved toward the symmetrical plane.

The parameters defined in Figure 2 reveal that glide symmetry is achieved when $a_{1}=b_{1}$, $a_{2}=b_{2}$, $a_{3}=b_{3}$, $a_{4}=b_{4}$, θ=−ϕ, and d=p/4.

**Figure 3:**
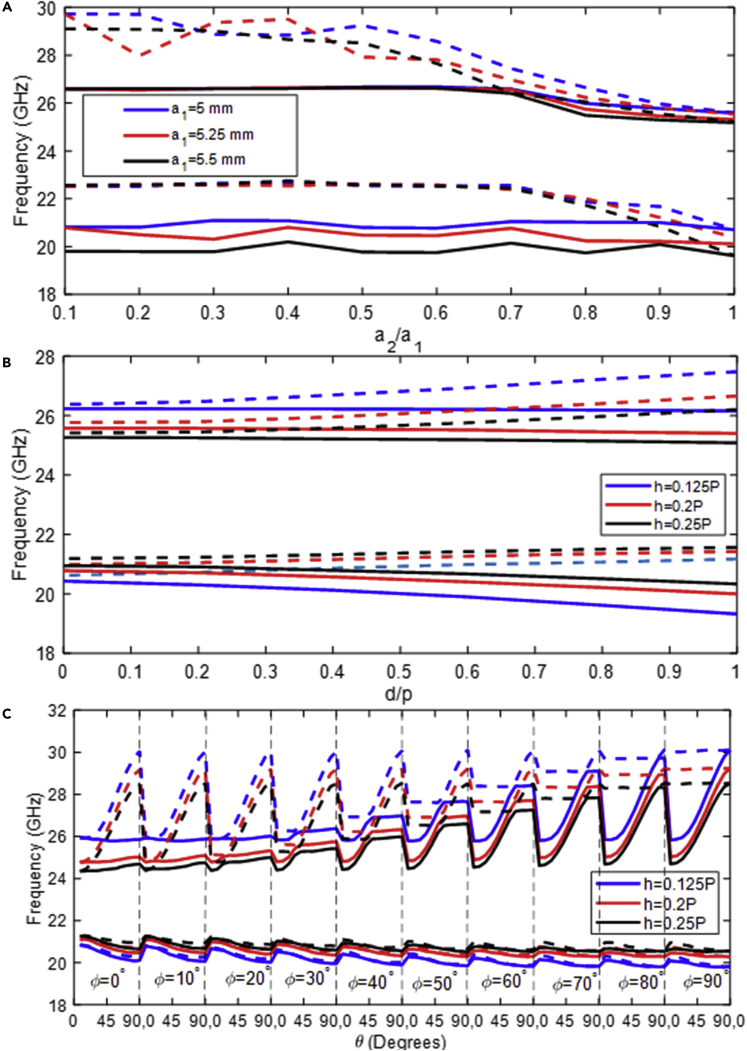
Analyzing the broken glide symmetry structure (A) The effect of the major radius of parallel ellipses versus minor radius of parallel ellipses ($a_{2}/a_{1}$) for various values of $a_{1}$. (B) The effect of displacement of parallel ellipses toward single ellipse (d/p) for different values of substrate thickness (h). (C) The effect of variation of angles θ and ϕ versus substrate thickness h. The parameters of the structure are: $a_{1}=b_{1}=5\;mm$, $a_{2}=b_{2}=2\;mm$, $a_{3}=b_{3}=5\;mm$, $a_{4}=b_{4}=3\;mm$, d=3.25mm, $\varepsilon_{r}=2.2$ and h=0.8mm.

**Figure 4:**
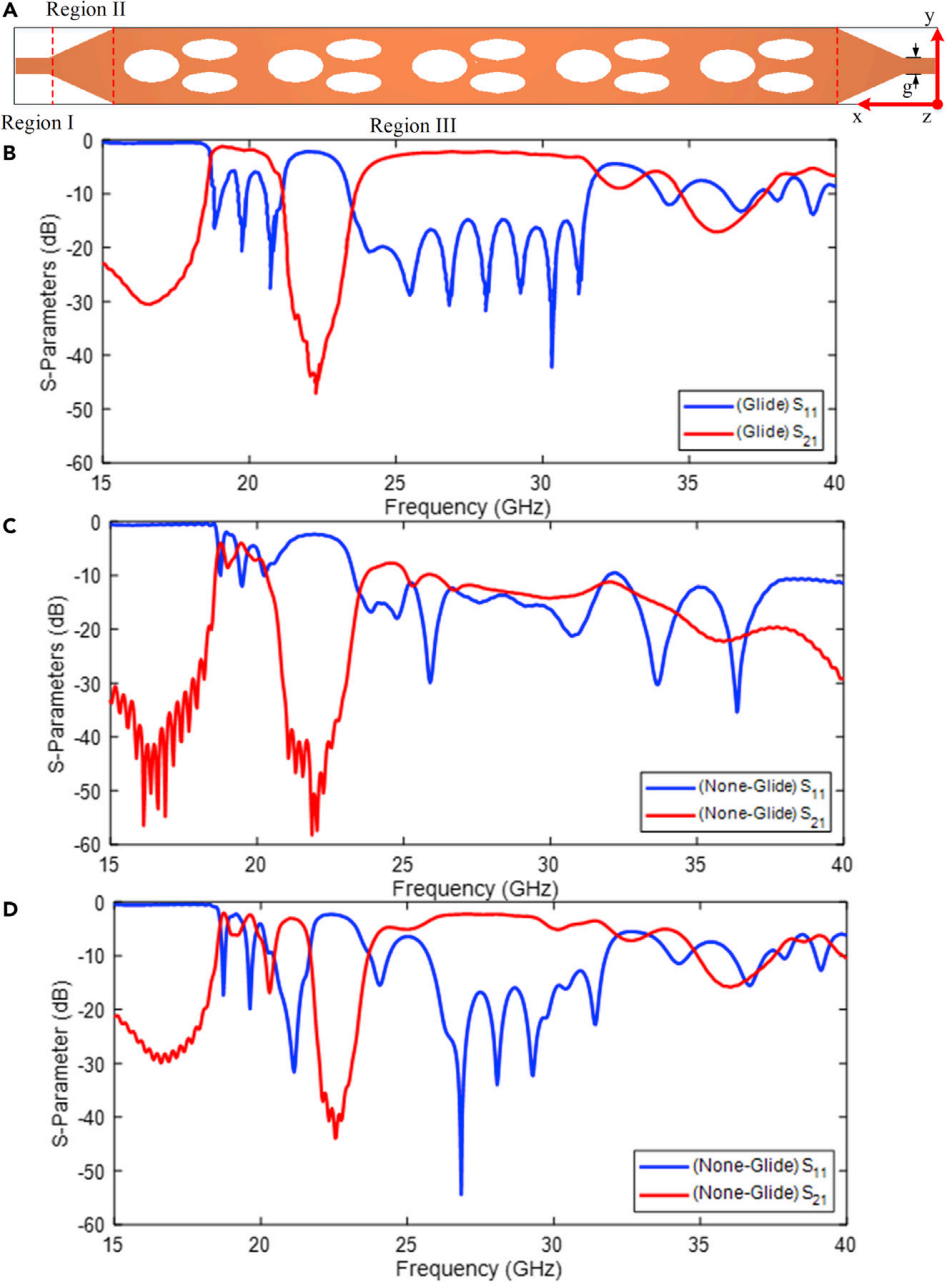
The presented glide symmetry filter (A) The detailed scheme of the designed glide symmetrical dual band filter. Simulated results of (B) glide symmetry, (C) none-glide symmetry based on $\theta =-\phi =45^{\circ }$, and (D) none-glide symmetry based on $d_{1}=2.5\;mm$.

The glide symmetry is formed by reflecting the initial ellipse slots through its symmetry axis and then transferring them at *h* in the z-direction. The symmetry axis passes through the center of the structure in the y-direction. Figure 2C- 4d provides an illustration of how this structure functions. The dispersion diagram of the proposed glide symmetrical structure is shown in Figure 2C. It is noted that Figure 2D illustrates the dispersion behavior of the structure broken by altering $d<d_{1}$. All dispersion analyses in this work were performed using the Eigenmode solver in CST Microwave Studio, with periodic boundaries in the x- and y-directions and magnetic boundaries in the z-direction. It should be noted that methods for quickly analyzing glide symmetry based on the Floquet theorem^37^^,^^38^ or equivalent circuits^12^ are available. As the axis of glide symmetry is y-directed, the phase variation is displayed in the mentioned direction. The parameters chosen for the case depicted in Figure 2C has the following dimensions: $a_{1}=b_{1}=5\;mm$, $a_{2}=b_{2}=2\;mm$, $a_{3}=b_{3}=5\;mm$, $a_{4}=b_{4}=3\;mm$, $\theta =-\phi =30^{\circ }$, d=3.25mm, $\varepsilon_{r}=2.2$, and h=0.8mm. Non-dispersive modes are obtained for a glide configuration (Figure 2C). The main advantage of breaking symmetry (Figure 2D) is the avoidance of electromagnetic field propagation in the desired frequency bands. As a result, the rejection-bands will be apparent. The symmetry rupture generates rejection-bands for the dispersion diagram value $\beta_{x}\;p/\pi =1$, as expected from a periodic glide-symmetric structure with two periodicities.^10^ The higher and lower limitations of the rejection-bands are indicated as reference points (Figure 2D). In addition, the reference points are derived for x-directed boundaries with a value of $180^{\circ }$ difference. It is noted that the phase value is normalized to 1 regarding the periodicity *p*. These reference points could well be responsible for demonstrating the characteristics of rejection-band and the refractive index in the parametric study described in Sections 3.1–3.3. The ratio of β and $\beta_{0}$ at different frequencies yields the effective refractive indices $n_{eff}$,

(Equation 6) $$n_{eff}=\frac{\beta }{\beta_{0}}$$

According to Equation 6, The refractive index increases as the frequency of the mode propagating in the medium under investigation reduces to lower values. The frequencies for the reference points at $\beta_{x}p/\pi =1$, with p=13mm in this section are 25.3 GHz, 24.8 GHz, 19.7 GHz, and 20.8 GHz. The tructure of Figure 2C is utilized as a reference for the parametric study ($a_{1}=b_{1}=5\;mm$, $a_{2}=b_{2}=2\;mm$, $a_{3}=b_{3}=5\;mm$, $a_{4}=b_{4}=3\;mm$, $\theta =-\phi =30^{\circ }$, d=3.25mm, $\varepsilon_{r}=2.2$ and h=0.8mm).

Three major subsections make up this section, each of which shows a distinct strategy for destroying symmetry and widening rejection-bands. The ellipse size ratio $\left(a_{2}/a_{1}\right)$, the horizontal displacement of the two parallel ellipses on the top layer normalized with regard to the periodicity (d/p), and the rotation of the single ellipses with respect to their coordinates are three essential parametric relationships that can be changed to disrupt the symmetry. The reference points specified on the dispersion diagram are represented by all graphs, as seen in Figure 2 ($\beta_{x}p/\pi =1$). The dashed lines represent the upper limit of the rejection-band, whereas the continuous lines represent the lower limit.

#### Breaking the symmetry using ellipse size and displacement variations

The first observed symmetry rupture occurs when the sizes of the parallel ellipses on the top and bottom layers differ. The relationship between the minor and major semi-axes is kept constant for simplicity ($b_{2}\;/b_{1}=a_{2}/a_{1}=0.4$). In Figure 3A, The rejection-bands open when the ellipses' minor radius, $a_{2}$, approaches zero whereas the size $a_{1}$ remains constant. It should be noted that for values less than $a_{2}/a_{1}=0.72$ and $a_{2}/a_{1}=0.45$, the first and second rejection-band widths begin to reach saturation. This is generally understood to mean that low profile ellipses are not suggested in acquiring a high rejection-band, which simplifies manufacturing. The glide symmetry has been regained, and the rejection-band has been totally closed for $a_{2}/a_{1}=1$ ratio, as expected. In addition, results for various sizes of parallel ellipses on the bottom layer ($a_{1}$ from 5 *mm* to 5.5 *mm*) are included. The structure’s periodicity *p* was kept unchanged at 13 *mm*. It is easy to see how shrinking the size of these parallel ellipses results in narrower rejection-bands.

The second symmetry rupture is caused by the displacement of the parallel ellipses on the top layer with the period, d/p. As d/p value increased, the symmetry rupture increases (see Figure 3B), and so do the rejection-bands. The significant effect of descending the displacement parameter has on the widening of the rejection-bands is of particular interest. This behavior is attributed to the fact that the effect of the elliptical structures becomes more noticeable as the field becomes more confined within the dielectric substrate. This also explains why, as the refractive index increases, the frequency shifts to lower frequencies for smaller values of the parameter. Furthermore, as the substrate thickness increases for the second band, the frequencies shift upward.

#### Breaking the symmetry using orientation angle variation of ellipses

A further method to break the symmetry is to vary the angles at which the top and bottom layers' single ellipses are orientated in opposite directions. Figure 3C depicts the behavior of the rejection-bands at θ angle of $0^{\circ }$ to $90^{\circ }$ for ϕ values ranging from $0^{\circ }$ to $90^{\circ }$ in steps of 10. To get a sense of how all of the structure’s parameters interact, see Figure 3C shows the results for various values of the dielectric substrate thickness normalized to the periodicity. It can be found that the curves in both frequency bands cross when θ and −ϕ are equal, which occurs when glide symmetry exists. As a result, the rejection-bands are open for θ values that differ more from ϕ.

In terms of dielectric substrate thickness, Figure 3C shows that the higher and lower limitations of the second frequency band increases. Furthermore, the first frequency band is nearly saturated. Thus, for the first frequency band, the mode is attenuated enough that an increase in thickness has almost no effect on it.

### Glide-symmetric dual band filter design

At microwave frequencies, a bandpass filter based on the system with glide symmetry described above is designed to assess the simulation results of the glide symmetric elliptical slots by the commercial software of CST Microwave Studio. The schematic scheme of the presented filter is depicted in Figure 4A. Following are the main three sections to the filter. Region I is a microstrip feed line structure that couples electromagnetic energy to the slot line on the top and bottom layers of the dielectric substrate, as indicated by the dotted red line in Figure 4A. The taper in Region II is designed to match the incoming electromagnetic energy to the glide symmetrical unit cells.

For the simulation demonstration, three designs were performed: glide, none-glide (breaking with displacement, case I), and none-glide (breaking with orientation angle, case II). The unit cells have the following dimensions: $a_{1}=b_{1}=5\;mm$, $a_{2}=b_{2}=2\;mm$, $a_{3}=b_{3}=5\;mm$, $a_{4}=b_{4}=3\;mm$, d=3.25mm, $\varepsilon_{r}=2.2$, and h=0.8mm, which are printed on Rogers 5880 dielectric substrate. The parameters of the designs differ: $\theta =-\phi =0^{\circ }$ (glide symmetry), $\theta =-\phi =45^{\circ }$ (breaking with orientation angle), d=3.25mm (glide symmetry), and d=2.5mm (breaking with displacement).

The glide configuration’s center frequencies for two passbands are 19.748 GHz and 28.233 GHz, as shown in Figure 4B. In the first passband, the insertion loss (IL) is around 0.83 dB and the return loss (RL) is less than −20 dB, according to the results. In the second passband, the simulated results show that the minimum IL is around 1.3 dB and the RL is around −32 dB. As illustrated in Figures 4C and 4D in none glide configurations, the return loss decreases (case I and case II). Furthermore, none glide configurations result in insertion losses of 5 dB and −3′dBat the first bandwidth, respectively.

**Figure 5:**
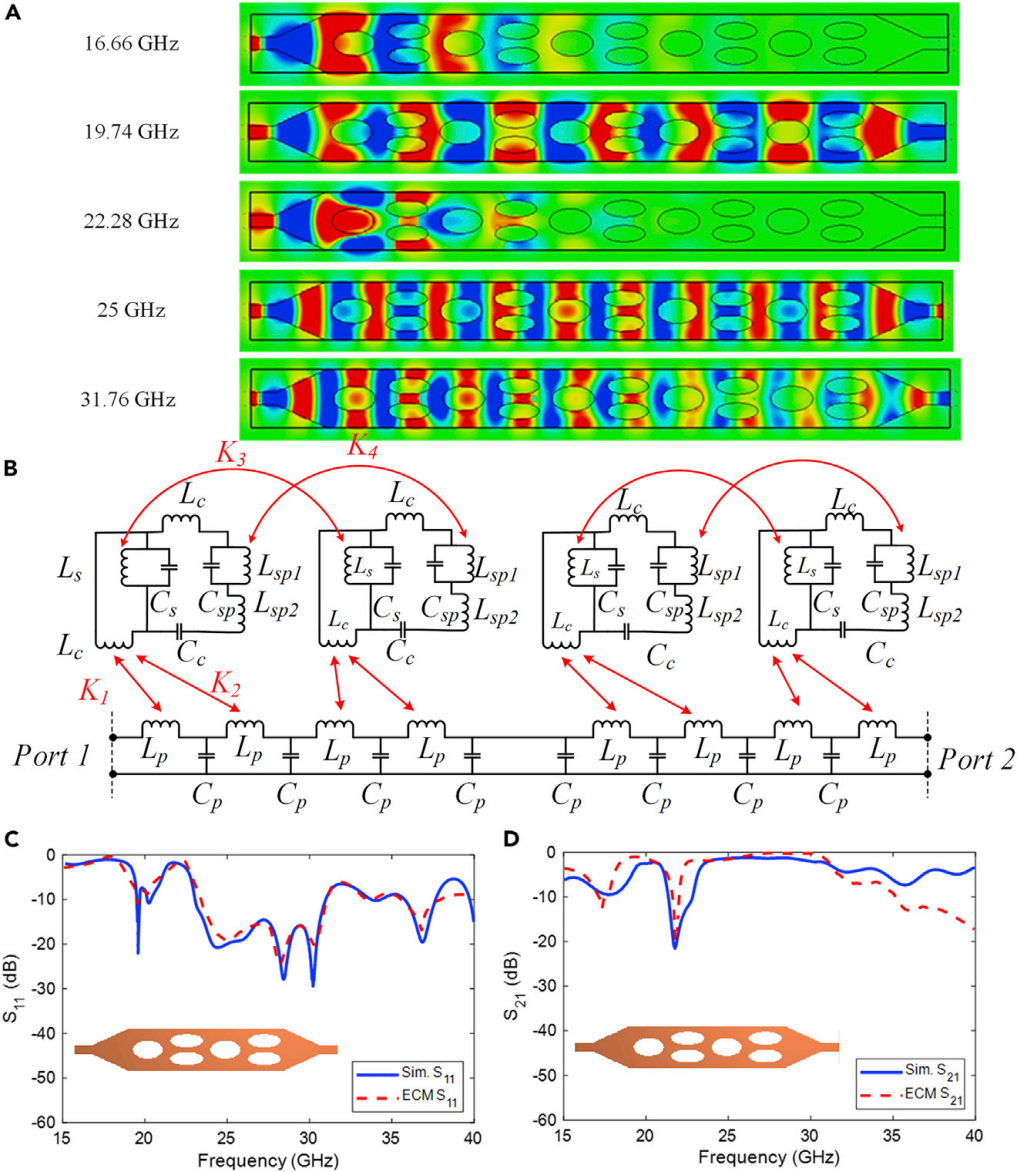
Analyzing the presented filter using current distribution and equivalent circuit (A) The simulated results of electric field distribution ($E_{y}$) on the glide symmetrical filter at the frequencies of interest. (B) Equivalent circuit model of the presented structure with two unit cells combining two parallel elliptical and two single elliptical slots. The values of the different elements for this case are: $L_{c}=0.4\;nH$, $C_{c}=0.6\;pF$, $L_{s}=4\;nH$, $C_{s}=0.27\;pF$, $L_{sp1}=0.36\;nH$, $C_{sp}=2.15\;pF$,$L_{sp2}=1.25\;nH$,$L_{p}=0.69\;nH$, $C_{p}=1.12\;pF$, $K_{1}=0.42$, $K_{2}=0.5$, $K_{3}=0.51$, $K_{4}=0.96$. The simulated and equivalent circuit model responses of the presented glide symmetrical structure. (C) $S_{11}$ and (D) $S_{21}$.

The simulated electric field distribution ($E_{y}$) on the x-y plane of the glide symmetric structure at 16.66 GHz, 19.74 GHz, 22.28 GHz, 25 GHz, and 31.76 GHz are plotted in Figure 5A to further obtain the propagation characteristics of the presented filter, respectively. The frequencies are conveniently selected to illustrate the most fascinating phenomena seen in this structure, including the appearance of the rejection-band (16.66 GHz), the maximal field attenuation obtained in the rejection-band (19.74 GHz), propagation between the rejection-band (22.28 GHz), the maximal attenuation obtained in the rejection-band (25 GHz), and propagation at a frequency higher than the rejection-band (31.76 GHz). The fields propagated uniformly in the glide arrangement, with the exception of the capture at 16.66 GHz and 22.28 GHz, where two rejection-bands were shown.

To gain a physical understanding of the effect of glide symmetry on the frequency response of the structure, we investigate the equivalent circuit model for the structure with glide symmetry. Figure 5B shows the equivalent circuit model for the glide-symmetric case. This structure corresponds to two periods of the initial structure, one in the top layer and one in the bottom layer, plus a translated half period, where the added length accounts for the translation performed in the glide-symmetric case. Each elliptical slots are represented as inductance-capacitance (L-C) circuit shunt. The single elliptical slot is indicated as $L_{s}-C_{s}$ whilewhereas the combination of parallel elliptical slots modeled as shunt $L_{sp1}-C_{sp}$ which in series with $L_{sp2}$. It is noted that there is magnetic coupling between the elliptical slots on the top and bottom layers. The coupling can be modeled as $K_{i}\;;i=1,2,3,4$ . It is assumed that the rectangular patch can be presented as $L_{p}$ and $C_{p}$ combination which is indicated in Figure 5B. The transmission response of this network is computed using the commercial software ADS. The circuit response is compared in Figures 5C and 5D with simulations of the same structure obtained with CST Microwave Studio. There is an acceptable qualitative agreement for the frequency range where two bandpass behavior appears at 19.80 GHz and 26 GHz (the frequency range of interest in this work).

**Figure 6:**
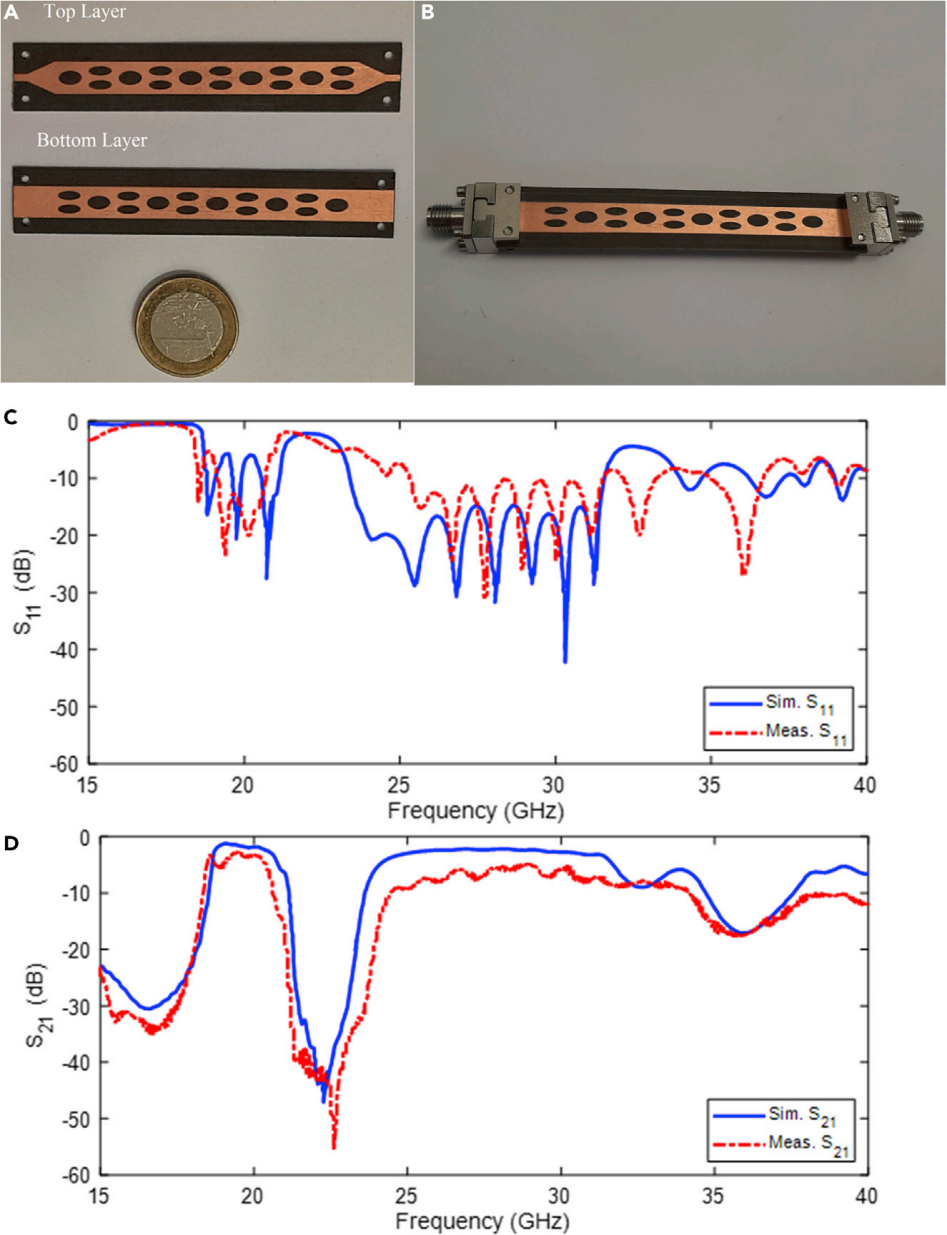
The measurement procedure (A) Top and bottom view of the presented dual band frequency response. (B) Photograph of the fabricated glide symmetry unit cell with 5 period. The simulated and measured results of the proposed glide symmetrical structure with 5 unit cells. (C) $S_{11}$ and (D) $S_{21}$.

To demonstrate the improved characteristics of glide symmetry in presented filters, a bandpass filter with 5 periodic cells, i.e., 5 pairs of elliptical slots etched on the top layer of the substrate but translated p/2 in the xaxis on the bottom layer, was designed. Taper transitions from the microstrip to the glide symmetrical surface were designed and optimized.^42^ The filter parameters for achieving the desired frequency response (center frequency, bandwidth, and rejection-band) have been designed directly. Figure 6 depicts the measured response of this glide-symmetric filter. The measured results show that in the first passband with a center frequency of 19.74 GHz, the 3 dB fractional bandwidth is approximately 12.33% and the IL is approximately 1.3 dB. The measured results show that the 3 dB fractional bandwidth in the second passband with a center frequency of 25 GHz is approximately 33% and the minimum IL is approximately 3 dB.

### Conclusions

This work proposed a model made up of glide symmetry concept according to single-plane metal structure. The utility of these structures lies in their ability to generate rejection-bands with a braided glide symmetric unit cell in a simple and straightforward implementation. The unit cell is loaded with elliptical slots that are glide symmetrically etched on Rogers 5880 dielectric substrate. Furthermore, broken glide symmetry provides an additional degree of freedom, allowing for the design of multiple pass and rejection-band filters, which can be used to suppress higher harmonics, for example. For the glide configuration, the experimental results revealed two passbands with bandwidths of 2.5 GHz and 8.26 GHz, centered at frequencies of 19.74 GHz and 25 GHz (12.33% and 33% fractional bandwidth). The structure’s adventure lies in its wideband feature. The low-cost filter can be integrated into antennas, lowering the overall profile. Filter designed with glide symmetric topology is capable to work beyond 100 GHz (6G communication band, for instance). The main advantageous of these structures are low loss within broadband bandwidth. In 6G technology, glide symmetry can easily be combined with other design concepts to improve various performance aspects. Moreover, robustness and cost effective of gap waveguide technology seem to be account for utilizing this technology in glide symmetric topology over 6G communication band. Consequently, these structures can be an appropriate alternative to traditional topology which confronts the fragility arising from the low profile of the components at high frequencies.

### Limitations of the study

The proposed filter based on glide symmetry principle can be employed in high frequency range. The two main reasons behind this are: (1) The filter needs at least two unit cells to be designed, and (2) the equivalent circuit model of the presented structure should be precisely to get desired responses. Owing to the feed line scheme, the filter requires to be matched to the input port. Actually, the proposed structure is sensitive to the feed line design.

## STAR★Methods

### Key resources table

**Table:** 

REAGENT or RESOURCE	SOURCE	IDENTIFIER
**Software and algorithms**		

CST Studio Suite	Dassault Systèmes	CST 2018
MATLAB	The MathWorks, Inc	R2019a

**Other**		

Vector Network Analyzer	Rohde&Schwarz	ZVA40

### Resource availability

#### Lead contact

Further information and requests for resources should be directed to and will be fulfilled by the lead contact, Mohammad Alibakhshikenari (mohammad.alibakhshikenari@uc3m.es).

#### Materials availability

This paper did not generate new unique reagents.

### Method details

#### Numerical analysis

All the numerical analysis required for this work was conducted by CST Design Studio Suite 2021. Firstly, an oval slot was designed which its dispersion diagram was analyzed. The single oval slot was then modified through glide symmetry principle. The dispersion diagram of the modified unit cell was discussed in terms of minor and major radius of the oval slots. Moreover, the rotation of the single oval affected on the bandwidth of the presented dual band filter.

#### Prototype fabrication

To get insight of the mechanism of the proposed glide symmetrical filter, 5 periods of oval slots unit cell was designed and fabricated. In order to match the input signal to the presented dual band filter, a taper transition was applied. In is noted that the structure is printed on a Rogers 5880 dielectric substrate.

#### Experimental measurements

In order to measure the characteristics of the proposed dual band filter, vector network analyzer (Rohde&Schwarz ZVA40) was used. The filter was connected to the vector network analyzer using high frequency cable. S- parameter ($S_{11}$ and $S_{21}$) was eventually measured.

## Data Availability

• Data reported in this paper will be shared by the lead contact upon request. • This paper does not report original code.
